# The childbearing health and related service needs of newcomers (CHARSNN) study protocol

**DOI:** 10.1186/1471-2393-6-31

**Published:** 2006-12-26

**Authors:** Anita J Gagnon, Olive Wahoush, Geoffrey Dougherty, Jean-François Saucier, Cindy-Lee Dennis, Lisa Merry, Elizabeth Stanger, Donna E Stewart

**Affiliations:** 1 School of Nursing, McGill University, 3506 University St., Montreal, Quebec H3A 2A7, Canada; 2 Department of Obstetrics and Gynecology, McGill University Health Centre, 687, Pine Ave. West, Room F2.27, Montreal, QC H3A 1A1, Canada; 3 School of Nursing, McMaster University, 1200 Main Street West, 2JRec. Rm 2J34a, Hamilton, ON L8N 3Z5, Canada; 4 Faculty of Medicine, Pediatrics, McGill University, 2300 Tupper Street, Rm A216, Montreal, Quebec H3H 1P3, Canada; 5 Division of General Pediatrics, MGill University Health Centre, 2300 Tupper Street, Rm A216, Montreal, Quebec H3H 1P3, Canada; 6 Department of Psychiatry, Centre hospitalier universitaire de mère enfant, L'Hôpital Sainte-Justine, 3180 Ellendale Avenue, Montreal, QC, H3S1W3, Canada; 7 School of Nursing, University of Toronto, 155 College Street, Toronto, ON, M5T 3M7, Canada; 8 School of Nursing, McGill University, 3506 University St., Montreal, Quebec H3A 2A7, Canada; 9 Department of Obstetrics and Gynecology, McGill University Health Centre, 3506 University St., Montreal, Quebec H3A 2A7, Canada; 10 Vancouver Coastal Health, 2733 Heather Street, Heather Pavilion – Room B213, Vancouver BCV5Z 1M9, Canada; 11 University Health Network, University of Toronto, 200 Elizabeth St., EN-7-229, Toronto, ON M5G 2C4, Canada

## Abstract

**Background:**

Refugee and asylum-seeking women in Canada may have significant harmful childbearing health outcomes and unmet health and social care needs. The most vulnerable of these women are: those who have left their countries by force (e.g., war, rape or abuse histories), are separated from their families, have limited knowledge of the host country languages, and are visible minorities. Asylum-seekers face additional stresses related to their unknown future status and are marginalized with regards to access to provincial health care systems. The prevalence and severity of health issues in this population is not known nor is the extent of response from social service and health care systems (including variation in provincial service delivery). Understanding the magnitude of health and social concerns of newcomers requires data from a representative sample of childbearing refugee and asylum-seeking women resettling in Canada to permit comparisons to be made with non-refugee immigrant and Canadian-born women. Our research questions are: (1) Do refugee or asylum-seeking women and their infants, experience a greater number or a different distribution of harmful health events during pregnancy, at birth, and during the postpartum period than non-refugee immigrant or Canadian-born women? (2) Are the harmful health events experienced postpartum by asylum-seeking women and their infants, addressed less often (compared to refugees, non-refugee immigrants, and Canadian-born women) by the Canadian health care system as delivered in each of the three major receiving cities for newcomers?

**Methods/design:**

This is a four-year multi-site prospective cohort study (pregnancy to 4 months postpartum). We will seek to recruit 2400 women [200 in each of 4 groups (refugees, asylum-seekers, non-refugee immigrants, and Canadian-born) from 1 of 12 postpartum hospital units across the 3 largest receiving cities for newcomers to Canada – Montreal, Toronto, and Vancouver].

**Discussion:**

Knowledge of the extent of harmful health events occurring to asylum-seeking, refugee, immigrant, and Canadian-born women, and the response of the health care system to those events and group differences, if they exist, will inform immigration and health policy makers as well as providers of services.

## Background

The U. N. definition of refugee: "owing to a well-founded fear of being persecuted for reasons of race, religion, nationality, membership in a particular social group, or political opinion, is outside the country of his nationality, and is unable to or, owing to such fear, is unwilling to avail himself of the protection of that country [1]" is being used in this study. Unfortunately, much of the literature in this field does not distinguish between refugees and non-refugee immigrants, rather "immigrants" are discussed as a whole. When possible, the distinction between immigrants with different migration histories (refugees versus non-refugee immigrants) has been presented separately here. Individuals seeking the protection of a country other than their own ("asylum-seekers") will, generally speaking, be successful in obtaining this protected status if they have histories similar to those individuals meeting the U.N. definition of refugee.

### Literature review

#### Refugees and asylum-seekers in Canada

There are a number of distinct classes of migrants to Canada. When examined together, migrants are multi-ethnic, their mother tongue and the language they use vary, and they have a variety of religious traditions, lifestyles, and political alliances. As opposed to "refugees," "independent-" or "family-class immigrants" choose to come to Canada and are motivated to leave their countries and re-establish themselves in a new country in the hope of a better life [2]. Their migration is planned and they are able to return to their countries of origin if they choose. On the other hand, "refugees" are, by definition, forced to leave their countries to ensure their survival. Their arrival in Canada is in many respects involuntary and they are not able to return to their countries of origin. All migrants will go through phases of adjustment but the permanent, forced nature of the refugee migration experience makes their integration into society more difficult [3,4].

Currently, there are close to 13 million refugees and asylum-seekers (applicants for refugee status) of concern to the United Nations High Commissioner for Refugees (UNHCR) [5]. Canada's role in supporting refugees dates back to its signing of the 1951 UN 'Convention Relating to the Status of Refugees.' In this role, Canada received 531,417 refugees (with an immigration total of 3,576,298) from 1979–1999, at a rate of about 24,000 per year in the last five years [6,7]. About 30,000 applications for asylum are received yearly in Canada [7-9]. The top ten source countries for refugees in 2000 were Yugoslavia, Sri Lanka, Afghanistan, Iran, Pakistan, the Democratic Republic of Somalia, India, Iraq, the Republic of Zaire, and Bosnia-hercegovina [9]. Croatia, Algeria, and the Democratic Republic of Sudan were among the next five [9]. These same countries have been among the top fifteen source countries since 1998, comprising 58% of refugees to Canada [9].

#### General refugee and asylum-seeker health

Immigrants to Canada (and the US) are healthier than the population living in the new country although they lose this advantage over time [10-13]. Immigrants must meet certain health and other criteria to be accepted into Canada and have enough economic resources to immigrate, likely resulting in a "healthy immigrant" effect. During our preparatory work, studies that addressed the health of migrants in Canada were identified [[10,14-19] Montpetit,[20-25]]. Of these, those that examined refugee health focused on general health [22-24] and mental health [15-18]. **No study was identified which specifically addressed reproductive refugee health in Canada. **Those studies focusing on reproductive health reported on migrants as a whole; results related to women refugees to Canada were not reported separately [14,19-21,25].

The general health of refugees to Canada was described in two studies. The first, conducted in Calgary, sheds light on *the health of government-sponsored refugees in Canada*. Health problems identified most often in the 1,104 government-sponsored refugees arriving in Calgary in the early 1990's included upper respiratory tract infections, 17.8%, impaired vision, 15.4%, dental emergencies, 12.8%, ear infections, 7.4%, gynaecological problems, 6.1%, and obstetrical conditions, 5.6%. Twenty-two (5.8%) required emergency hospitalization [24]. Thus, even in refugees receiving medical screening prior to entering Canada, several health issues were identified. This may be due to superficial medical screening in the source country or a lengthy period of time between initial exam and arrival in Canada or both [24]. The second study, conducted in Montreal in 1985/86, sheds light on *the health of those seeking asylum in Canada at the border or once in the country *(as opposed to government or NGO-sponsored refugees arriving via consular offices overseas) [22]. Of the 1,994 applicants who received a medical examination at a clinic identified by the Quebec government as the care provider for refugee applicants, 87% were considered to be in good health, 3% had a major handicap or chronic illness, and 10% were in poor health. Health problems identified included nutritional deficiencies, stunted growth, anaemia, parasitic infections, syphilis, inadequate vaccination, evidence of physical torture, and risk for tuberculosis. More recent refugee groups present a similar health profile with malnutrition, dehydration, gastrointestinal problems, scabies, head lice, minor skin conditions, and hepatitis B [26].

The rather large amount of published literature on torture and abuse suggests that an increasing proportion of morbidity and mortality is the result of widespread human rights abuses [27,28]. Physical consequences of torture may include: general organ systems damage, psychosomatic, neurological, and musculoskeletal symptoms, subcutaneous fibrosis and compartment syndrome of the feet, skin changes, scars, organic brain syndrome, and sexual dysfunction; psychological consequences include post-traumatic stress disorder, generalized anxiety, major depression, psychosis, and substance abuse [29]. A review of the mental health of refugees and immigrants to Canada [15], based largely on the experiences of Southeast Asian refugees, showed that exposure to catastrophic stress such as torture and rape leads to post-traumatic stress disorder (PTSD). Unemployment was suggested as being an important problem in refugees to Canada since refugees admitted for compassionate reasons may not have employable backgrounds [30]. Extended separation from family members is also suggested to have a harmful psychological impact [31]. As compared to other immigrants, refugees have been found to have a higher rate of somatization, somatic symptoms without a clear physical aetiology such as headache, abdominal pain, low back pain, dizziness, and insomnia [32].

#### Refugee and asylum-seeking women's health

There are several factors that appear likely to be key in considering the health of women refugees to Canada. These include a history of torture) [33-42]and sex and gender-based violence (SGBV; includes rape, sexual assault, and sexual harassment and abuse) [15,33-35,39-41,43-46], female genital mutilation (FGM) [20,47], migration history [14,22,24,39,40,44,48,49], somatization [32,50], post-traumatic stress disorder (PTSD) [33,35-37,40,51-54], and official language difficulties [14,22,23]. Increased risk of infectious diseases [22,24,39,40,43,44,48,55] and poor maternal nutritional status [40,56] are seen in women who have spent time in refugee camps and in war-torn areas [46].

Included among the determinants of health outlined by Health Canada are gender and culture [57], nonetheless pre- and post-migratory experiences remain under-explored as factors related to women's health. Refugee women face added obstacles to maintaining their health and well being compared with Canadian-born women [58,59]. Language barriers, isolation and loss of pre-existing social support systems, pre-migratory losses, long family separations, ruptures and traumatic events constitute vulnerability factors that affect the already fragile refugee women's health [58,60,61,61]. Refugee women have often cited experiencing rape, sexual abuse, harassment and/or the obligation to grant sexual favours in return for food or necessary papers before or during their migration process, which exacerbates post-traumatic disorders. Additional problems face them during resettlement resulting in a perception of marginalization from mainstream society [34].

Female genital mutilation is performed in several source countries of refugees to Canada including countries in Africa, Southeast Asia, the Middle East, and Central and South America [47]. There are several types of procedures [62]. In addition to the chronic health effects of these procedures including urinary tract infections and painful menstruation, difficulties arise during labour and birth [20,63]. The most severe form, infibulation, is estimated to occur in 99% of all Somalian women and 85% of those from the Sudan [20], two of the top fifteen source countries for refugees to Canada [9].

Refugee and asylum-seeking women of childbearing age will interact with the system around the time of birth when other, previously unknown health issues may be found. One of the few reports of pregnant women seeking asylum in Canada was from a clinic in Buffalo, New York. In a group of 59 women, urinary tract infections, monilial infections, scabies, head lice, otitis media, intestinal parasites, vaccine-preventable infections, low pregnancy weight gain, anaemia, short inter-pregnancy spacing, history of inadequate health care, and female genital mutilation prohibiting pelvic examination were found [44]. Several of these women had witnessed deaths and torture of family members, and rape. Depression was common. The extent to which these health issues, or others, occur in pregnant refugee women Canada-wide has yet to be determined. Childbearing health issues that affect all women regardless of immigration status must be considered. Determinants and effects of low birth weight, illness prevention and health promotion activities are among the several variables to consider. Space constraints prohibit discussion of these.

#### Provincial variation in service provision to refugees and asylum-seekers

Classes under which individuals migrate to Canada determine which health and social services are available to them. Broad immigration classes include "independent" (autonomous workers), "family" (family members), "refugees" (based on the UN definition above), and "asylum-seekers" (those applying for refugee status at the border or in the country). Individuals arriving with permanent residence status, usually in the "independent" or "family" categories are eligible, after a 3-month wait, for the same health and social services as those available to Canadians. Health and social service coverage for refugees and asylum-seekers, however, varies. Unlike others in Canada, refugees may not be covered by provincial health plans upon arrival in Canada and asylum-seekers are not eligible. In its place, the Interim Federal Health Program (IFH) may be available to them. This program was put in place for humanitarian reasons to allow refugee claimants, Convention refugees, and others under immigration control to receive essential health care. It is not meant to replace provincial health plans and does not provide the same extent of coverage. Eligibility for this program is determined by a demonstrated lack of funds, which is assessed by an immigration officer and this, only once refugee status has been confirmed or eligibility to make a refugee claim has been approved. **Despite the existence of the Interim Federal Health Program, certain asylum seekers have no health insurance for periods of time due primarily to delays in processing their requests to apply for refugee status. **Health and social service provision during this waiting period varies by availability of services through local non-governmental organizations and community health centres. "Inland" claimants (i.e., those seeking asylum once in Canada) have the most difficulty. We asked for feedback from refugee service providers (via the Canadian Council for Refugees (CCR) electronic mailing list) on variation in eligibility for services by province. One respondent reported that in Montreal, the usual delay is three weeks (personal communication – D. Isaacs), while in Ontario refugees can wait up to one year to obtain coverage (personal communication – South Etobicoke Community Legal Services, Toronto). Port-of-entry (i.e., at the border) asylum-seekers could receive the necessary forms as they enter the country or up to one month after arriving and declaring their intention to make the claim. If there are problems with their "Notification of Intention to Make a Refugee Claim" form, it can be returned to them and delays occur in getting their IFH coverage. Newborns of refugee claimants and Convention refugees born in Canada are eligible for IFH until eligible for provincial health coverage with some exceptions in British Columbia and Ontario. Further, many service providers are unfamiliar with the IFH forms and will not provide services to those covered by it for this reason (personal communication – P. Dongier). This suggests that health care coverage for refugees and asylum-seekers and services received by them are anything but uniform and the rate at which they have access to services differs from one province to the next. No such difference in health care coverage has been reported between immigrants from other categories and individuals born in Canada. **A systematic examination of the response of the health care system to specific needs of newcomers by province has not, to our knowledge, been made.**

### Study framework

**Figure 1:**
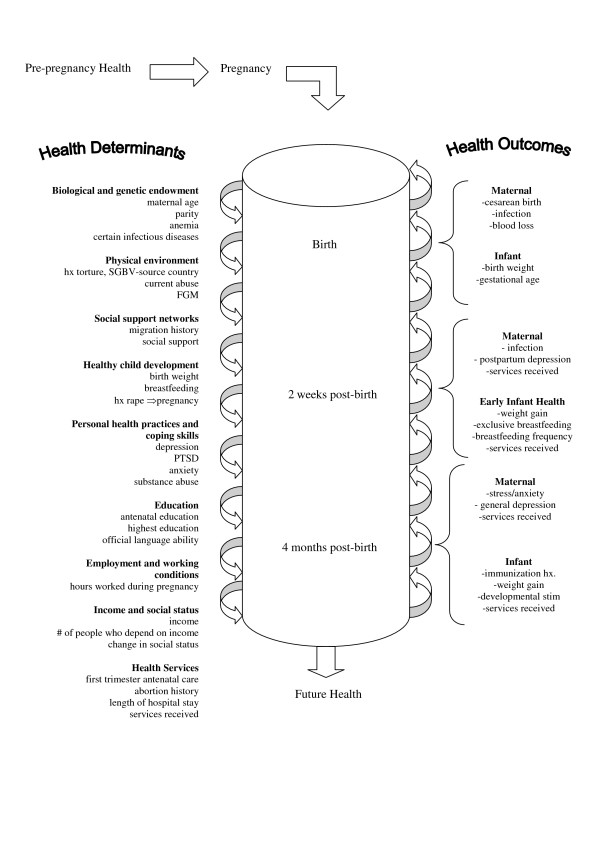
Pregnancy health chart.

The foundation for the work of our group is the current understanding of health determinants as described in The Ottawa Charter on Health Promotion [64]. The Ottawa Charter has been translated into over 40 languages and serves as a basis for policy and action around the world [65,65]. Categories of health determinants are based on the work of the Federal, Provincial, and Territorial Health Ministers in 1994 [66]. They include: income and social status, social support networks, education, employment and working conditions, physical environment, biology and genetic endowment, personal health practices and coping skills, healthy child development, and health services [66,67]. Each of our projects applies the framework of the Ottawa Charter from a different angle or with a different focus, while simultaneously considering the remaining aspects; health outcomes at one point may be health determinants at another (see figure 1).

### Summary of background

Little is known about the reproductive health of women in Canada as it relates to their migration history. Most reports are of all migrants combined, with inadequate numbers of refugees or asylum-seekers to speak authoritatively about their health. Studies that do address the health of refugees or asylum-seekers are generally small, and do not focus on reproductive health. One of the few studies of asylum-seekers to Canada suggests that pregnant asylum-seeking women are likely to have significant health needs. Refugee and asylum-seeking women play a vital role in meeting Canada's international humanitarian objectives and their special health care needs can only be addressed with adequate data. While health determinants are generally known, their distribution in childbearing refugee and asylum-seeking women is not, and whether the distribution differs from that of women with other migration histories is also not known. Despite the potentially greater vulnerability of refugee and asylum-seeking women and their infants, the provision of care to this population around the time of birth has been under-studied. Virtually no published data currently exist about this potentially significant area of unmet health need.

### Research questions

Our research questions are: (1) Do refugee or asylum-seeking women and their infants, experience a greater number or a different distribution of harmful health events during pregnancy, at birth, and during the postpartum period than non-refugee immigrant or Canadian-born women? (2) Are the harmful health events experienced postpartum by asylum-seeking women and their infants, addressed less often (compared to refugees, non-refugee immigrants, and Canadian-born women) by the Canadian health care system as delivered in each of the three major receiving cities for newcomers?

## Methods/design

### Overview of the design

Two thousand four hundred women speaking one of the 13 study languages [Arabic, Dari/Persian, English, French, Mandarin/Cantonese (oral; 'simple' and 'complex' Chinese written), Punjabi, Russian, Serbo-Croatian, Somali, Spanish, Tamil, and Urdu] together with their 2400 or more infants will be recruited on the postpartum units of hospitals serving a high percentage of immigrants in cities receiving the greatest number of refugees (Toronto, 32.8%; Montreal, 21%; and Vancouver, 7.7%) [9] and followed for four months. Women will be divided into one of four study groups – refugees, asylum-seekers, non-refugee immigrants, and Canadian-born according to their migration history and status in Canada. Since there will be more non-refugee immigrant and Canadian-born women available, we will alternate recruitment of these women based on closest date and time of birth to each refugee or asylum-seeking woman identified. Inclusion criteria and language capabilities will be determined through interviewer-assisted questionnaires administered on the postpartum unit by research assistants with the help of interpreters if needed. Health data from the index pregnancy, labour, and birth, will be obtained from medical records; study inclusion criteria, general health information, discrimination experience, and breastfeeding plans will be obtained by interviewer-assisted questionnaires administered on the postpartum unit. Breastfeeding frequency, infant weight gain, postpartum depression, social support, abuse, presence of health concerns, and services use will be assessed at two weeks postpartum during a home visit by a nurse (with interpreters, if needed). Health risk assessment, general infant health, breastfeeding duration, post-traumatic stress disorder (PTSD), anxiety, depression, services use, migration history, and presence of health concerns will be measured at four months postpartum during a second home visit by the same nurse (with interpreters if needed). The nurse will be blind to the study research questions; determination of wellness will be based on standard criteria normally employed by nurses during home visits in the early postpartum period [68,68-72]. Mother-infant dyads for whom the nurse identifies no concerns at the time of the visit will be considered to have had their concerns addressed. Mother-infant dyads for whom the nurse identifies concerns and for whom insufficient services have been provided or arranged by the time of that visit (as assessed by an independent nurse expert), will be considered to have 'unaddressed concerns'. Verification of data inconsistencies will be immediately identified and corrections requested at the source. The NAC, CAC and liaison advisory groups will provide counsel for all study aspects. This research will be carried out in accordance with the Helsinki Declaration and the appropriate institutional ethics approval has been obtained.

### Study population

We will recruit women at the time of birth in health care facilities normally frequented by newcomers (see Table 1). Identifying refugee women either through their obstetricians'/family practitioners'/midwives' offices or at their first trimester ultrasound was also considered. However, we felt that many women might be overlooked for participation given that the data available on immigrant women generally suggests health services under-utilization [73] and the single report identified of pregnant refugee women en route to Canada noted that they received health care late in their pregnancies [44]. Recruitment on the postpartum unit has been successful in the past and has been put forward as an acceptable and valid approach to consider for population-based research [74]; this procedure was successfully used in our previous work.

**Table 1:** Canadian Hospitals Identified for CHARSNN Study Recruitment

**Hospital**
*Montreal*
Jewish General
St. Mary's Hospital Centre
Lasalle Hospital Centre
Sacré Coeur Hospital
St. Luc Hospital
McGill University Health Centre

*Toronto*
Humber River Regional Hospital (×2 sites)
Scarborough General Hospital
St. Joseph's Health Centre
St. Michael's Hospital
Toronto East General Hospital

*Vancouver*
Children's and Women's
Simon Fraser Health Region
Providence Health Care

#### Inclusion criteria

*All*: Planning to remain in the study city until four months postpartum, able to speak any one of the thirteen study languages [Arabic, Dari/Persian, English, French, Mandarin/Cantonese (oral; 'simple' and 'complex' Chinese written), Punjabi, Russian, Serbo-Croatian, Somali, Spanish, Tamil, and Urdu], lives within 15 kilometres of hospital, discharged by day 4 postpartum for vaginal births or day 7 for caesarean births, fits into one of the categories below (see additional file 1). *Refugee*: Women meeting the U.N. definition of refugee within the last five years, "owing to a well-founded fear of being persecuted for reasons of race, religion, nationality, membership in a particular social group, or political opinion, is outside the country of his nationality, and is unable to or, owing to such fear, is unwilling to avail himself of the protection of that country [1]" (this category includes UNHCR channelled refugees as well as women or their spouses who applied for refugee status after arrival in Canada and received a positive response). *Asylum seeker:* Women who have applied or are in the process of applying for refugee status in Canada within the last five years, and those with no known current immigration status. *Non-refugee immigrant*: Women who immigrated within the last five years with non-refugee history (determined by migration history). One will be recruited for every alternate refugee/asylum seeker. *Canadian-born*: One will be recruited for every alternate refugee/asylum seeker.

#### Exclusion criteria

*Maternal:* Major hearing impairments, major mental illness (schizophrenia, other psychoses, or profound previously existing depression), or cognitive impairment that precludes giving fully informed consent; holds a valid 'Visitor' Visa or claims to be a visitor. *Infant:* Planned antepartum to have the infant adopted, and stillbirth or infant died.

#### Recruitment procedures

The same recruitment procedures as those successfully used in a previous study are proposed here. An information sheet briefly describing the study and offering women the possibility of declining any visit from research staff will be given to mothers upon admission to the hospital (or posted in the hospital room, depending on the wishes of the hospital staff). Research assistants will explain the study to eligible women (who do not decline their visit) on the postpartum units. Women with limited official language capabilities often have a bilingual family member with them for their waking hours in hospital however, research assistants will contact appropriate interpreters by telephone if needed or requested by the women. In that case, the interpreter will explain the project to the woman by telephone (using a translated standardized information sheet). The research assistant will obtain consent for participation and baseline data via the interpreter. We are aware that literacy levels may be inadequate for as many as 15% of potential refugee/asylum seeking participants [9]. We are confident that we can assist all these women by using appropriately translated questions and interpreters as is currently being done. Reasons for non-participation and basic descriptive data will be recorded for all women declining participation if the woman willingly provides this information, and these data will be compared to participants. Reasons for potential study subjects being excluded from the study will be documented. Research assistants, to determine if possibly eligible women are on the unit and to maintain interest in the project, will visit postpartum units daily. We obtained ethical approval for 4–5 hospitals in each of the three cities in our previous project and are effectively recruiting in all cities using the procedures outlined here.

#### Availability of study subjects

Data from our previous project, using identical inclusion/exclusion criteria as those defined here, were used to assess availability of subjects for study. Overall, our previous experience showed that we were able to categorize women by migration history and recruit them at a reasonable rate. We will further optimize our recruitment rates in the proposed study by focusing recruitment in hospitals with the highest recruitment rates, and by hiring research assistants full-time to permit more time to be spent on each of the postpartum units (e.g., in Montreal 11.4% of eligible subjects were lost because they were discharged before they could be approached). We estimate study loss between consent and 1^st ^home visit to be 30% and loss between 1^st ^and 2^nd ^home visit to be from 10% – 15% and we will therefore recruit 2400 women to achieve a final sample of 1440.

It is possible that recruitment rates for any one of the four groups may differ from the others (e.g., in Montreal, recruitment rates in the asylum-seeking group were greater than in the refugee group) and that rates per group will differ by city. This will be carefully monitored, as was done in the previous study. In the case of extreme (>25%) differences in group' sizes, adjustments will be made in the order of recruitment (e.g., 2 women to the same group may be recruited rather than alternating, as is currently planned) to maintain groups at a similar size as much as possible. This was not previously required. Potential group imbalances have been considered in sample size calculations (see below). The momentum from previous studies (e.g., familiarity with hospital staff, etc) will optimize recruitment for the proposed study. Our previous studies show that subjects are available and willing to participate to permit us to answer our research questions.

### Data intake

#### Description of the measures

Health determinants and their outcomes, consistent with the population health promotion framework upon which this study is based, will be measured (see figure 1). Baseline, pregnancy, and birth-related data will be measured on the postpartum unit by medical record review and questionnaire, known to be successful for the type of data being sought [75,76] and found successful in our previous project. Follow-up data will be collected at 2 weeks and 4 months postpartum in the home. Up to three telephone contacts will be made to obtain follow-up data and to minimize losses. This broad examination of childbearing refugee health is needed given the general paucity of existing population-based data to support health research and policy priorities in childbearing refugee and asylum-seeking women. Recall, there are no provincial health service datasets that exist for an important percentage of our study population since they are not eligible for health service reimbursement by provincial plans and the IFH database is not yet available for general research use. Questionnaires were revised using stringent procedures [77-80]. Other measurement strategies previously found to be successful will also be employed such as baby weight in the home [81]. Most questions requiring greater sensitivity are scheduled for the final visit, at 4 months, by which time a relationship is more likely to have been established between the nurse and the woman.

Health system response to concerns will also be measured. "Unaddressed concern", is defined as a nurse-identified concern (based on standards for postpartum care [68,70,71,82-85]) with either the woman or her infant at two weeks and/or four months post-birth (see Tables 2 &3), for which no professional attention is being given or is planned. If there are no concerns, the family will be considered to have all concerns addressed [85]. These assessments have been conducted successfully by project nurses in the home in the early postpartum period in previous work [86]. Those concerns that appear to be more subjective such as psychosocial concerns have been made as objective as possible by applying standardized assessment criteria. Care provided or arranged during the first four months post-birth will be obtained via maternal report (diary) of care received. These reports will be completed with assistance from the nurse at the 2-week home visit, 2 months (via telephone at the time of the first follow-up telephone call) and at the 4-month visit to ensure they are complete. Diaries of care are being successfully used in all 13 languages in our previous work and were used in other studies [86,87].

**Table 2:** Potential concerns to be assessed at 1–2 weeks postpartum by a project nurse in the home

Infant *Feeding:* • Infant weight (< birth weight) • < 3 wet diapers in 24 hrs • < 1 stool in 24 hrs • Infant given other food/fluids other than breast milk or formula • Infant not receiving vitamin D supplements *Safety:* • Sleeping prone (↑ risk of Sudden Infant Death Syndrome) • Infant rides in car without car seat *Infection:* • Purulent discharge from eyes or conjunctivitis *Hyperbilirubinaemia:* • Jaundice visible below the umbilicus *General health:* • Blood in stool • Persistent vomiting and/or black/green/red in colour and/or copious amounts (> 250 mls) of vomit in the last 24 hours *Psychosocial:* • Insufficient clothing (as measured by < 1 change of indoor clothing, inappropriate outdoor wear, < 1 blanket)	Maternal *Pain:* • Uterine → visual analogue scale (VAS) pain score > 4 in past 24 hrs (based on WHO Pain Ladder) • Perineum → VAS pain score > 4 in past 24 hrs • Breast (general) → VAS pain score > 4 in past 24 hrs • Breasts (engorgement) → VAS pain score > 4 in past 24 hrs • Nipples → VAS pain score > 4 in past 24 hrs • Back pain → VAS pain score > 4 in past 24 hrs *Bleeding:* • Soaking > 3 pads per 24 hrs • Clotting *Breast care:* • Pain while breastfeeding → VAS pain score > 4 in past 24 hrs • Pain prohibiting breastfeeding *Infection:* • Perineal discharge *General:* • Blood pressure (diastolic >90 systolic < 90 mm Hg) *Psychosocial:* • Signs of depression (score ≥ 10 on the Edinburgh Post-natal Depression Scale [91]) • Absence of social support (as measured by the Personal Resources Questionnaire – Q 11 [71] • No knowledge of tel. #911 in case of severe health problem or if in danger (self or infant) • Inadequate food (i.e., Woman has missed or reduced meals because of lack of resources to buy food)

**Table 3:** Potential concerns to be assessed at 4 months postpartum by a project nurse in the home

Infant *Feeding & Growth* • Weight <10^th ^percentile for age or weight decreased 20 percentile points [71] • Not receiving Vitamin D supplements [92] • Cow milk is being given [92] *Safety* • Blind cords or other unsafe objects within infant's reach of where infant sleeps • Infant sleeps prone • Unsafe crib (space between bars > 6 cm, plush mattress) • Unfinished bottles with curdled milk lying in area where infant sleeps • Car-seat positioned front-facing • Smoke exposure *Development* • Infant unable to do two or more of the following: turn head side to side to follow a toy, glance from one object to another, turn head toward a sound, make sounds in response to people/looking at toys, respond (i.e., face brightening) to people's voices, laugh and smile, move arms and legs in response to being spoken to, finish feeding within 45 mins, lift head and support self on forearms, bring hands to chest and keep head in midline when lying on back, hold head steady when supported in a sitting position, reach for an object, hold an object briefly when placed in hand *General Health* • Bowel movements abnormal (blood in stool; no BM for > 5 days) • Bruising or wounds or burn marks or evidence of healing bruises, wounds or burn marks on infant's body • Persistent rash (i.e., broken skin or blisters for > 2 weeks) • Abnormal vital signs (at rest – heart rate >150/min, respirations >45/min, indrawing or increased respiratory effort) • Signs of dehydration including drowsiness, dry mouth, darker than normal urine, sunken fontanel. *Immunization* • No 2 month vaccination (DPT-Hib-Polio) • No 4 month vaccination (DPT-Hib-Polio) & no planned appointment *Psychosocial:* • Insufficient clothing (as measured by < 1 change of indoor clothing, inappropriate outdoor wear, < 1 blanket)	Maternal *Pain *(as measured by VAS pain score > 4/10 in past 24 hours) • Abdominal • Breast or nipple • Back pain • Hemorrhoids • Perineal • Other pain *General Health *[93] • Any faecal or urinary (as measured by self-report) • Urinary tract infection (urgent, painful, frequent urination) • Perineal infection or STI (vaginal discharge with or without foul odor) • Woman has not received all of her immunizations *Psychosocial* • Inadequate food (i.e., Woman has missed or reduced meals because of lack of resources to buy food) • No knowledge of tel. # 911 in case of severe health problem or if in danger (self or infant) • Unaddressed birth control needs (as measured by Health Risk Assessment) • Signs of abuse (measured by the Abuse assessment form [94] and as suspected by nurse) • Signs of depression (score ≥ 10 on the Edinburgh Post-natal Depression Scale [91]) • Signs of depression, somatization or anxiety related to trauma (as measured by Hopkins Symptom Checklist) • Signs of PTSD (as measured by Harvard Trauma Questionnaire)

For all concerns, professional attention may be given/planned with a nurse, midwife, or a medical doctor. In the case of breastfeeding difficulties, professional attention may also be given/planned with a lactation consultant; and in the case of psychosocial concerns, professional attention may also be given/planned with a social worker or psychologist. Professional attention is generally justified for these concerns since they are considered the standards for care. We recognize that the presence of one or more unaddressed concerns may not lead to morbidity and that the family's lay support system may also be involved in resolving concerns however, our principle interest is in the response of the health and social system to these concerns.

All project nurses' records and all corresponding mothers' reports of care will be reviewed by an independent nurse expert based in Montreal (blind to the research question) to determine if concerns were identified and if they were addressed or planned to be addressed. Migration history, language and other information to suggest the mother may be a refugee or an asylum-seeker will be removed from the record prior to her/his assessment. In the case where the independent nurse expert is unable to make the determination, that set of records will be adjudicated to unanimity by an expert panel consisting of a nurse, a medical doctor (obstetrician-gynaecologist and a paediatrician or a family physician attending births), and a social worker. We anticipate approximately 15% of all subjects, 216 mother-infant pairs, to require adjudication. If, in a small number of cases, the panel can not reach unanimity, the case will be considered indeterminate and sensitivity analyses will be conducted to assess the impact of these cases on the results.

#### Recruitment, activities, and training of staff

A city co-ordinator will be recruited for each of the 3 cities to oversee the day-to-day operation of the study in those cities; another, national coordinator will be hired to co-ordinate the entire study across cities. Research assistants will recruit subjects and collect data with interpreters as necessary. Project nurses will collect outcome data with the assistance of interpreters as necessary. "Test" records will be used to complete the record review forms to ensure agreement across research assistants. Case studies will be used to orient research assistants and project nurses and to ensure standardization of recording across families. Nurses will also receive training in response strategies to use with victims of psychological trauma.

#### Quality assurance procedures

The national coordinator, in conjunction with city coordinators, will ensure quality content through standardized procedures. The person responsible for collecting the data will correct identified errors as soon as possible. In the case where the family is no longer in hospital and a home visit has not yet been made, the project nurse will obtain missing data. Data missing from the project nurses' records will be obtained by telephone or repeat home visits. Our previous project showed rates of missing data to be low for all questionnaires, except the Migration and Resettlement Questionnaire as administered in Montreal because recruiters, in an effort to be efficient, often left the questionnaire to be completed by the woman independently while they recruited other women. This procedure is being changed in the proposed study, such that the questionnaire will be administered at the 4-month home visit by nurses.

### Analysis

#### To respond to research question #1

All data will be sent to the Study Coordinating Centre at the McGill University Health Centre – Royal Victoria Hospital Site for analysis once data from the last subject has been obtained and data verification procedures have been completed. The analyses planned include: **(1) Descriptive statistics **for each health determinant and health event of interest during pregnancy, birth, and 2 weeks and 4 months postpartum (including rates of concerns) for women in each of the study groups. In addition, chi-square test and ANOVA will be employed to examine the balance of the baseline characteristics between the study groups.**(2) Bivariate analyses **to test the relationship between each independent variable and each outcome, using either the Cochrane-Mantel-Haenszel test (to calculate the odds ratios, relative risks), T-test, or ANOVA, depending on the nature of the variables. **(3) Multivariate analyses **to examine the effect of group on pregnancy, birth, and 2-week and 4-month outcomes. Multiple linear regression or logistic regression models will be used to explore the relationship between the outcomes and the variables of interest. **(3a) **Variables identified as statistically significantly related to the outcome at the .10 level and any others known *a priori *to be strong predictors of the outcomes but not reaching the .10 level will form the maximum model. The Bayesian Information Criterion (BIC) will be used in the model selection procedure. The BIC will be used to create a series of the most plausible regression models, given the data. We will examine all models produced for the presence of confounding by looking for relatively large changes in coefficients following exclusion or inclusion of various combinations of independent variables. The most clinically relevant variables – based on the estimates of effect and their 95% confidence intervals for the independent variables included in the model – will be selected for inclusion in the final regression model. **(3b) **Group status (refugee, asylum-seeker, non-refugee immigrant, Canadian-born) will be forced into each model. **(3c) **Multivariate analyses will be repeated for each outcome separately. **(3d) **Model assumptions will be validated through examination of residuals in the case of multiple linear regression and linearity of the logit in the case of logistic regression. **(4) Hierarchical regression modelling **to examine effects within province. This is a multi-province, multi-centre study. Differences in the health system may be present between centres within provinces and between provinces leading to clustering effects. Hierarchical regression models allow the incorporation of variables measured at different levels of a hierarchy and accounts for correlation within clusters. Moreover, one can estimate the parameters separately within each province to permit comparison with more pooling power [88,89].

#### To respond to research question #2

"Unaddressed concerns" has been previously defined (see above, Description of the Measures). We will estimate the proportion of each group with each unaddressed concern and calculate 95% confidence intervals for proportions using standard techniques to permit population inferences to be made. We will compare the proportion of each group with unaddressed concerns by calculating the difference in proportions in each group and the 95% confidence intervals for the difference based on standard statistical techniques. We will compare the proportion of women with unaddressed concerns in each of the provinces through logistic regression procedures with province and group forced into the model. 95% confidence intervals for odds ratios will be calculated based on these regression results. We will also use hierarchical regression modelling to gather additional information to respond to this research question, with techniques similar to those used to answer research question #1 (above).

#### Exploratory analyses

Exploratory analyses will be conducted to identify any potential 'very vulnerable' sub-groups of women and infants, those at greatest risk of harmful health outcomes and unaddressed concerns at both 2 weeks and 4 months postpartum. Variables to be considered in these analyses will include socio-economic status, social support network, and length of time in Canada, to name a few. Our focus will be to identify similarities among those who either had a great number of harmful health outcomes or unaddressed concerns or in whom there was a persistence of harmful health outcomes or unaddressed concerns over the 4-month period.

### Sample size considerations

Our primary interest is in testing whether refugee or asylum-seeking women are at more risk than non-refugee immigrants or Canadian-born women in experiencing harmful health events during their pregnancy, at birth, and during postpartum. To detect a difference of 10 percentage points at a power of 80% and a significance level of 0.05, we require 275 women in each study group (thus a total of 1,100). For events with non-rare exposure rate (≥10%), we will be able to detect odds ratios of 1.5, using logistic regressions, at 80% power and at 0.05 significance levels, with a sample of 1,434. Therefore, our final sample of 1,440 women will be adequate as regards these analytic strategies. For rare exposure events, we will have less power using logistic regression models; however, by stratifying the analysis for the three provinces, variation is likely to be reduced due to possible correlation within clusters, and thus the power will be increased. The statistical package PASS 2002™ was used in sample size calculations [90]. These calculations were made using the prevalence figures and expected differences for 'events' from preliminary Montreal data from an earlier project and for 'unaddressed concerns' from the same dataset. We are aware that less stable estimates of inter-provincial differences may be obtained if group sizes differ by province. We will therefore aim for similar group sizes by province. However, we estimate that in any province, with a minimum of 100 women in each group and an event rate of about 20%, we will be able to accommodate 8 explanatory variables in multivariate models, a reasonable number for the analyses planned.

### Control for bias

#### Sample distortion

Research assistants will visit birthing centres caring for a high percentage of newcomers daily to ensure potential study subjects are not missed. Selecting non-refugee immigrant and Canadian-born women who give birth at the closest date and time of the woman recruited to the refugee or asylum-seeking group ensures representativeness of our population of interest for each of the study groups. Postpartum, women will be contacted up to three times by the project nurse to ensure they are not lost to follow-up.

#### Information

Rigorous validation procedures were used for the questionnaires to be employed here thus maximizing equivalency across all language groups on questions being asked. Data from each comparison group will be obtained similarly. Training of project nurses will ensure consistency of approach and will be confirmed by repeating outcome data collection for 5% of the sample.

#### Confounding

Data on all known potential confounders will be obtained. In the event of substantial confounding, multivariate analyses will be used to address these group differences. At the 2-week visit, nurses will be instructed to assess without providing care and to refer for serious 'unaddressed concerns' if needed rather than providing care themselves since this could bias study results at the 4-month contact. At the 4-month visit, nurses may respond to issues raised by the sensitive nature of some questionnaires since data collection will be complete at that point. Most sensitive questions are scheduled for the last visit to enable the nurse to respond to issues, which may arise without compromising study integrity.

## Discussion

### Ethical considerations

No subject may enter this study without having first signed the consent form. This will ensure that the subject understands the personal implications of participation and has voluntarily chosen to join. All information on participants will be held in strict confidence. Women will be reimbursed for time spent and inconvenience as detailed in the budget. Nurses will provide emergency care at the time of the visit, if they identify that it is needed, until the family can be referred to an emergency department. Planned ethical safeguards specific to the vulnerable nature of the study population of CHARSNN can be seen in Table 4.

**Table 4:** Planned Ethical Safeguards Specific to the Vulnerable Nature of theStudy Population of CHARSNN

**Ethical Guidelines**	**Planned Safeguards**
***Respect for justice & inclusiveness***	

*Tri-Council Guidelines *[95]*Article 1.1a. *All research that involves living human subjects requires review and approval by an REB in accordance with this Policy Statement, before the research is started... Justice connotes fairness and equity. Procedural justice requires that the ethics review process have fair methods, standards and procedures for reviewing research protocols, and that the process be effectively *independent*.	
*Tri-Council Guidelines *[95]*Articles 5.1–5.3 – Inclusion in research. *Principles of distributive justice reflects respect to human dignity and diversity. States that people should not bear unfair burden of research, nor be unfairly excluded (including exclusion on the basis of cultural, religion, race..., ethnicity..., unless there is a valid reason to do so).	NAC, CAC and ECLG formed and 13 study languages chosen in such a way to ensure maximum representation of target population in the 3 study cities. Categories of recruitment are based on migration status (with scientific support), and participants are recruited into one of four groups, distributing the burden of participation to promote knowledge of health of vulnerable populations and prevent exploitation of any one group.
*Recruiting uninsured populations *Not only should investigators seek to enroll uninsured, but they should focus research on medical and socially vulnerable areas, including effects on health of decreased access to care [96].	See above.
*Recruiting populations with precarious immigration status. *Refugees and asylum seekers are a vulnerable population, and utmost care must be taken not to coerce participation. However, it is unethical not to deal with problems and inequities facing refugee populations [97].	See above.

***Respect for free & informed consent***	

*Tri-Council Guidelines *[95]*Articles 2.1–2.8. Free and Informed Consent. *(e.g. potential subjects informed of study info, harms/benefits, voluntary, ongoing consent, etc). *Article 2.1(b). *Where written consent is culturally unacceptable, or where there are good reasons for not recording consent in writing, verbal agreement, or handshake can be used and should be documented. Provisions should be made that any language barriers should not disqualify any participant from informed consent.	Information about the study is available in all rooms before women are for approached the study. Women can request unit staff that they not be approached by a researcher. Back-translation and validation of the consent forms and all questionnaires/protocols have been done. Readability tests were conducted on all documents. All documents were reviewed by ECLG and monolinguals for cultural appropriateness and recommended changes made. Increased time is allotted for explanation of study and consent process in case of language, cultural barriers and to prevent any undue coercion to participate.
	The consent is left with the woman if she does not want to make her decision right away. Interpreters are used when needed. Women who give informed consent but prefer not to sign their name (for cultural reasons, concerns of confidentiality during immigration process) may give verbal consent. Where requested for cultural reasons, a husband can be the one to sign the actual consent form for a woman but only *after *she has given her *own *informed consent. Women are reminded at all contacts that they may withdraw at any point in the study process.
*Tri-Council Guidelines *[95]*Article 4 – Conflict of interest. *...consent must be voluntarily given, without manipulation, undue influence or coercion.	Researchers, recruiters and study nurses do not provide direct patient care unless a nurse identifies an emergency situation during a home visit (i.e., they are independently hired for the research study).

***Respect for privacy & confidentiality***	

*Tri-Council Guidelines *[95]*Articles 3.1–3.6*. Provisions must be made to *ensure privacy and confidentiality*. This includes special care with secondary use of data and creation of data linkages.	All data are strictly confidential. All nurses and interpreters follow a strict code of confidentiality. Recruiters and data entry clerks sign confidentiality forms when hired. All data are coded and summarized to prevent identification of individuals in reports of results. No data linkages are being made. No personal info is communicated to CIC or other government agencies. No study results can have any impact on individual migration or welfare status as results are de-nominalized. All women are reassured about this, and this reassurance is restated in the consent form. Women who choose to have interpreters present have the option of choosing an interpreter who is *not *from their community (to further reassure women that information will not get back to their community).

***Respect for vulnerable persons***	

*Obtaining community input *Mistrust is an ethical challenge to recruitment. Can be improved with feedback from community and advisory boards [98,99].	NAC, CAC, and ECLG formed to assess cultural appropriateness and to find strategies to increase participation while decreasing potential harm or offence.
*Recruiting vulnerable populations. *Women having just given birth may be considered vulnerable. However, recruitment on postpartum units is an acceptable and valid approach [74]. Educating research team of ethical guidelines and basic rights of participants is necessary and will improve ethical quality [98].	There will be careful screening of recruiters and nurses who are hired. Two days of initial training will include sensitivity issues for recruiting on postpartum populations. Additional support will be offered by the research team to the women as deemed necessary. Reimbursement will be made to all women participating. Based on ECLG feedback, trained nurses are being used instead of non-nurse research assistants. Nurses at home visit will provide emergency care if needed, until referral to an Emergency Department is possible.
*Culturally appropriate approach to gathering data*	Interview-style or self-administration of questionnaires are offered to each woman. Extra time is accorded for the woman to ask questions, consider, consult husband, etc.

***Balancing harms & benefits***	

*Recruiting populations that may have been victims of trauma or oppression *It is misguided (even unguided) to argue that studies should not be done because of potential harm [100]. Research on refugee populations with traumatic experiences can have beneficial effects if carried out sensitively and appropriately.	Research nurses will receive *specific *1-day training by trauma professionals to provide response strategies for working with victims of psychological trauma. The team also includes expert trauma professionals, who are available for any individual referrals of women at risk and needing extra psychological support. NAC and CAC professionals in trauma offer additional support to the study procedures. Based on NAC, CAC and ECLG feedback, the burden of participation will be distributed over three encounters. Based on ECLG feedback, women will be explained reasons for asking sensitive questions. The most sensitive topics will be assessed at the final home visit with nurse, i.e. after more trust has developed between the women and the nurse.

***Minimizing harm***	

*Avoid, prevent or minimize harms *[95]	As described above several steps have been taken to ensure protocols and questionnaires are culturally appropriate. Sensitive questions are administered at the 2^nd ^home visit so participants are "warmed-up" to the study and have developed a relationship with the nurse. Introductory statements always precede sensitive questions. To minimize "questionnaire burden", questionnaires are administered over 3 interviews. To minimize inconvenience, nurses visit women in their homes.

***Maximizing benefit***	

*Dissemination of research findings*	Partnerships with NAC and CAC include health, policy and advocacy stakeholders. The strong multidisciplinary research team coupled with ongoing meetings with NAC and CAC facilitate representation and moral responsibility to the vulnerable study population. Diversity of stakeholders also suggests that findings will be disseminated to the largest possible audience in a position to make appropriate recommendations to health, community and government policies after the study is completed.

### Timeline

This study will be conducted over a 4-year period. *September 2004 – January 2005*: hold investigator-collaborator meetings (includes CAC meetings in the respective cities, NAC teleconferences, full-investigative team or steering committee teleconferences, and a face-to-face meeting of the investigative team), submit proposal for ethical review, finalize and translate data collection tools not yet translated, set-up data management system, refine study protocols and procedures, and hire and train staff. *February 2005 – January 2008*: hold investigator-collaborator meetings, recruit and collect data. *February 2008 – May 2008*: hold investigator-collaborator meetings, complete data collection and prepare programs for data analysis. *June 2008 – August 2008*: hold investigative-collaborator meetings, conduct analyses and write reports.

### Feasibility

We have a strong, multidisciplinary, team who previously conducted four funded studies in migration and reproductive health. Members are associated with clinical, health services research, and research dealing with societal/cultural influences on health. The team includes a nurse-epidemiologist, a psychiatrist, a pediatric epidemiologist, an anthropologist, a health administrator, a cultural-psychiatrist, a nurse researcher, a bio-statistician, and experts in health promotion and in equity issues. Each will contribute scientifically and be responsible for ensuring the smooth running of the study in their respective cities. A large database will be built from this study with central data management permitting us to manage the data efficiently. Adequate time has been planned to permit women to answer these questions, we have ensured that the burden of questioning will be spread out over the three interviews, and we have made provisions to reimburse women for their time. We have previously been successful using the same study design as that proposed here.

### Knowledge translation/exchange

**Figure 2:**
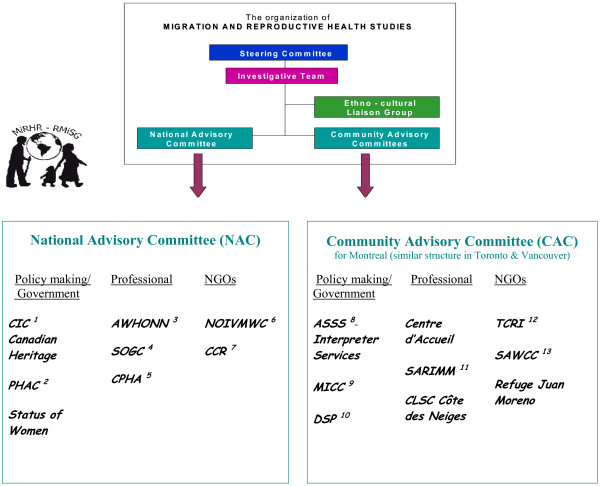
**Organizational Structure of Migration and Reproductive Health Studies**. 1. Citizenship and Immigration Canada (CIC); 2. Public Health Agency of Canada (PHAC); 3. Association of Women's Health, Obstetric and Neonatal Nurses (AWHONN); 4. Society of Obstetricians and Gynecologists of Canada (SOGC); 5. Canadian Public Health Association (CPHA); 6. National Organization of Immigrant and Visible Minority Women of Canada (NOIVMWC); 7. Canadian Council for Refugees (CCR); 8. Agence de la santé et des services sociaux Montréal (ASSS); 9. Ministère de la culture et des communications (Quebec) (MECC); 10. Direction de santé publique de Montréal (DSP); 11. Service d'aide aux réfugiées et aux immigrants du Montréal métropolitain (SARIMM); 12. La Table de concertation au service des réfugiés et des immigrants (TCRI); 13. South Asian Women's Community Centre (SAWCC).

Structured partnering with individuals from policy, practice, and research sectors, as well as with representatives of recipients of care is a special aspect of the program of research of which this project is a component. Evidence for this structure can be seen in the organizational layout of the program of research and in the terms of reference for the National and Community Advisory Committees (see additional files 2 and 3). Investigator, government, and non-governmental partner teleconferences are held at least twice yearly. Through this mechanism, the conceptual framework for our studies and the variables to be measured were finalized. Translation procedures were also reviewed, and plans to ensure optimal community involvement through the creation of ethno-cultural liaison groups were made. The organizational components of the program are summarized below (see figure 2).

### Implications/relevance

The reproductive health of refugee women is important for several reasons: the key role of foetal and infant development as a predictor of adult health, the role of newcomers to Canada in the development and maintenance of the society, and for humanitarian reasons. The reproductive health of refugee and asylum-seeking women is understudied and refugees have been identified as an important immigrant sub-group meriting investigation. The only approach to accurately quantify the distribution of health determinants and health care delivery differences in refugee, asylum-seeking, non-refugee immigrant and Canadian-born women in Canada, is through a large Canada-wide study. We propose to gather data on a final sample of 1440 women and their 1440 infants longitudinally over the first 4 months of the infant's life. National studies can be optimally conducted if informed by knowledge-exchange among key stakeholders and researchers, which our organizational structure facilitates. Results will be disseminated through the Metropolis Project; Metropolis' raison d'être is to promote research that is policy relevant – several of our team are members of the Metropolis Centre of Excellence in their city. Results will be further disseminated through the usual scientific forums as well as those suggested by our key stakeholders. We have used a scan of research commissioned by Health Canada to define refugee health as a priority research area and letters from Citizenship and Immigration Canada and others attest to the relevance of this project to them. In addition to providing data to drive policy, these results will form the basis for future studies which will examine priority health issues identified in this study in greater depth using a variety of methods, and test health and psychosocial support interventions for this population. Agencies represented on the NAC and CAC will make use of results from these studies in future planning both as it relates to health and social interventions and immigration policies, and in defining research agendas.

### Post-funding revisions to the protocol

Certain changes were made to the proposal after it was funded either in response to scientific reviewer or ethical review committees' suggestions, or to optimize feasibility or efficiency.

#### Recruitment strategy

Recruitment sites were revised based on a list of hospitals ranked according to greatest IFH reimbursement submissions which was provided to us by CIC after the study had been submitted for funding. Those hospitals with the greatest number of births to women likely to be eligible for our study have more intense recruitment – that is to say, each consecutive birth in those hospitals is being assessed for eligibility every day. This is the case for 2 to 3 hospitals per city. In those hospitals with fewer expected numbers of eligible births (according the IFH data), assessment of eligibility is being made for every consecutive birth but every 2–3 days rather than daily and only for women from source countries for refugees/asylum-seekers or who have IFH. Women who give either written or verbal consent are enrolled in the study.

#### Data collection

Certain questionnaires were reduced in length to reduce interview time for participants (e.g., the Discrimination Questionnaire was reduced, drug use questions were removed). Certain changes were made to the timing and frequency of administration of certain questionnaires: the Abuse Assessment Screen was moved to the 4-month visit and the EPDS is now administered twice, once at each home visit. Concerns being recorded by the nurse at the home visit (i.e., Tables 2 and 3) were reviewed and revised – included in the published protocol is the revised version.

#### Timeline

This study will be conducted over a 4-year period plus 5 months. *October 2004 – January 2006*: held investigator-collaborator meetings (includes CAC meetings in the respective cities, a NAC teleconference, full-investigative team teleconferences, and a face-to-face meeting of the investigative team), submitted proposal for ethical review at the administering institution and local Montreal/Toronto hospitals, finalized and translated data collection tools not yet translated, set-up data management system, refined study protocols and procedures, hired and trained staff and PI and national coordinator visited other cities and their local recruitment sites. **Note that the National Coordinator was on mat-leave for 9 months (i.e., Jan 2005–Sept 2005) which is the reason for this extended set-up period. *January/February 2006 – March 2006*: held one large face-to-face investigator-collaborator meeting, proposal submitted for ethical review to local Montreal/Toronto hospitals, recruitment began in Montreal and Toronto. *April 2006 – March 2008*: hold investigator-collaborator meetings, PI and national coordinator to visit other cities, ethical reviews and recruitment set-up to be finalized and data collection started across all cities, and begin preparation of programs for data analysis. *April 2008 – March 2009*: hold investigative-collaborator meetings, complete recruitment and data collection including the 'Expert nurse review' to determine 'addressed vs. unaddressed concerns', finalize data cleaning, conduct analyses and begin the write-up of manuscripts.

## Competing interests

The author(s) declare that they have no competing interests.

## Authors' contributions

AJG, the guarantor of this article, wrote the first draft of this manuscript, obtained funding, and was responsible for all aspects of the study. GD provided key expertise in defining the health outcomes and their analysis. All authors are co-investigators or collaborators on the study and contributed to its conceptualization. One author is responsible for implementation of the study in their city (OW in Toronto) and one is coordinating the study nationally (LM). We would like to acknowledge Becky Palmer for initially coordinating the study at the Vancouver site. All authors read and approved the final manuscript.

## Pre-publication history

The pre-publication history for this paper can be accessed here:



## Supplementary Material

Additional file 1 Appendix 1. CHARSNN Exclusion/Inclusion Criteria. Inclusion and exclusion criteria for CHARSNN study Click here for file

Additional file 2 Appendix 2.1. National Advisory Committee's (NAC) Terms of Reference. National Advisory Committee objectives, mandate, and composition Click here for file

Additional file 3 Appendix 2.2. Community Advisory Committees' (CAC) Terms of Reference. Community Advisory Committee objectives, mandate, and composition Click here for file
