# Supplementary material for: The childbearing health and related service needs of newcomers (CHARSNN) study protocol
Source: BMC Pregnancy Childbirth. 2006 Dec 26;6:31. doi: 10.1186/1471-2393-6-31 (PMC1797193; doi:10.1186/1471-2393-6-31)
Supplement: Additional file 1 — Appendix 1. CHARSNN Exclusion/Inclusion Criteria. Inclusion and exclusion criteria for CHARSNN study [file 1471-2393-6-31-S1.doc]

## CHARSNN Exclusion/ Inclusion Criteria

## Exclusion

# *Maternal*

- Major hearing impairments
- Major mental illness (schizophrenia, other psychoses, or profound pre-existing depression) or cognitive impairment that precludes giving fully informed consent
- Must be home for 3 days to be visited by the nurse (e.g. for women who deliver vaginally they must be discharged by day 4 and for women who have cesarean births, they must be discharged by day 7)
- Holds a valid visitor’s Visa or is planning on leaving Canada within 1 mo.

# *Infant*

- Planned antepartum to have the infant adopted
- Stillbirth or infant died

### Inclusion

- Must fit into one of the population categories described below
- Must live within a 30-45 minutes drive from hospital where they delivered
- Planning to live at the same address at the time of outcome assessment (i.e., 1-2 weeks postpartum and at 4 months postpartum) or to be at an address within the designated study area
- Able to speak one of the 13 study languages (English, Arabic, French, Serbo-croatian, Spanish, Mandarin or Cantonese, Punjabi, Tamil, Dari/Persian, Urdu , Somali, and Russian)

| Title | Refugee | Asylum Seeker | Immigrant | Canadian-born |
| --- | --- | --- | --- | --- |
| **Definition** | - Government or privately sponsored through UNHCR channels - Originally port of entry or inland claimants granted a positive response - Those ineligible/rejected claimants who were granted status on ‘humanitarian or compassionate grounds’ or accepted based on the pre-removal risk assessment - Spouse/dependent of any of the above - < 5yrs in Canada | - Port of entry claimants - Inland claimants - Those in pre-claimant status or those ineligible to make a claim (if the latter may be in process of applying to stay based on humanitarian or compassionate grounds) - Rejected claimants who are in the appeal process or applying to stay based on ‘humanitarian or compassionate grounds’ - Those who received a rejection to their claim or are ineligible to make a claim but are not deported because home country is on the moratorium list - Ineligible/rejected claimants undergoing a “Pre-removal risk assessment” - Those ineligible/rejected claimants on all grounds and awaiting deportation - No known immigrant status - Spouse/dependent of any of the above - < 5yrs in Canada | - Independent immigrants (economic or family reunification) - Students - Temporary workers - Provincial nominee or skilled worker program - Spouse of any of the above - Live-in Caregiver - < 5 years in Canada | - Born in Canada |
