# Supplementary material for: The childbearing health and related service needs of newcomers (CHARSNN) study protocol
Source: BMC Pregnancy Childbirth. 2006 Dec 26;6:31. doi: 10.1186/1471-2393-6-31 (PMC1797193; doi:10.1186/1471-2393-6-31)
Supplement: Additional file 2 — Appendix 2.1. National Advisory Committee's (NAC) Terms of Reference. National Advisory Committee objectives, mandate, and composition [file 1471-2393-6-31-S2.doc]

*
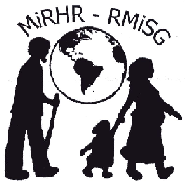
*

***Migration and Reproductive Health Research (MiRHR)***

#

*National Advisory Committee’s (NAC)*

# Terms of Reference

# Re-Approved – May 25, 2006

**Objectives:**

- To participate, from a national perspective, in obtaining scientific evidence to support the development of policies and programs which have the best outcomes for refugee and other mothers and their infants
- To ensure the dissemination of research findings at the national level
- To advocate and/or initiate changes in national policy and practice guidelines based on research results

**Mandate:**

The National Advisory Committee will contribute to these studies as follows:

- Assist with interpretation of national findings
- Advise on policy and practice implications of findings
- Advise on appropriate mechanisms for disseminating the findings
- Advise on relevant funding opportunities

**Composition:** Representatives from national immigrant serving agencies (NOIVMWC, other NGOs), professional health and other social service organizations (AWHONN-Canada, SOGC, CPS, CPHA etc.) and federal government departments (CIC, Public Health Agency of Canada, Canadian Heritage, etc.).

Meetings: 2 times per year (and at the discretion of the committee). In-person meetings every 2 years
