# Supplementary material for: The childbearing health and related service needs of newcomers (CHARSNN) study protocol
Source: BMC Pregnancy Childbirth. 2006 Dec 26;6:31. doi: 10.1186/1471-2393-6-31 (PMC1797193; doi:10.1186/1471-2393-6-31)
Supplement: Additional file 3 — Appendix 2.2. Community Advisory Committees' (CAC) Terms of Reference. Community Advisory Committee objectives, mandate, and composition [file 1471-2393-6-31-S3.doc]

*
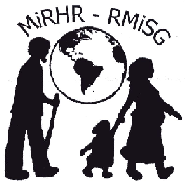
*

***Migration and Reproductive Health Research (MiRHR)***

#

# *Community Advisory Committees’ (CAC)*

# Terms of Reference

###### Re-Approved – May 25, 2006

**Objectives:**

- To participate, from a community perspective, in obtaining scientific evidence to support the development of policies and programs which have the best outcomes for refugee and other mothers and their infants
- To ensure dissemination of research findings at the community level
- To advocate and/or initiate changes in local policy and practice guidelines based on research results
- To advise the research team about local contextual factors that might affect the research environment

**Mandate:**

The Community Advisory Committees will contribute to these studies as follows:

- Advise on research procedures such as appropriate recruitment strategies
- Assist with formation of Ethno-Cultural Liaison Groups by suggesting the names of appropriate ethno-cultural community representatives to participate in this group and helping to maintain their interest in participating
- Assist with interpretation of local findings
- Advise on local policy and practice implications of significant findings
- Advise on appropriate mechanisms for disseminating the findings
- Advise on relevant funding sources

**Composition:** Three committees, one in each city (Montreal, Vancouver and Toronto) which include representatives from immigrant umbrella serving agencies (e.g. La table de concertation des organismes au service des personnes réfugiées et immigrantes, etc.), local health and social services providers (e.g. South Asian Women’s Community Centre, SARIMM, etc.), provincial and municipal government members (e.g. MSSS, MECC etc.) and the academic community.

**Meetings:** 2 times per year (and at the discretion of the committee)
